# The Compartmentalization of Amyloid-β in Idiopathic Normal Pressure Hydrocephalus Brain Biopsies

**DOI:** 10.3233/JAD-240167

**Published:** 2024-05-14

**Authors:** Sylwia Libard, Monika Hodik, Kristina Giuliana Cesarini, Anca Dragomir, Irina Alafuzoff

**Affiliations:** a Department of Pathology, Uppsala University Hospital, Uppsala, Sweden; b Department of Immunology, Genetics and Pathology, Uppsala University, Uppsala, Sweden; c BioVis Platform, Uppsala University, Uppsala, Sweden; d Department of Neurosurgery, Uppsala University Hospital, Uppsala, Sweden

**Keywords:** Alzheimer’s disease, Alzheimer’s disease neuropathological change, amyloid-β, idiopathic normal pressure hydrocephalus

## Abstract

**Background:**

Amyloid-β (Aβ) is one of the hallmark lesions of Alzheimer’s disease (AD). During the disease process, Aβ undergoes biochemical changes, producing toxic Aβ variants, proposed to be detected within the neurons. Idiopathic normal pressure hydrocephalus (iNPH) causes cognitive impairment, gait, and urinary symptoms in elderly, that can be reversed by a ventriculo-peritoneal shunt. Majority of iNPH subjects display different Aβ variants in their brain biopsies, obtained during shunting.

**Objective:**

To study the cellular compartmentalization of different Aβ variants in brain biopsies from iNPH subjects.

**Methods:**

We studied the cellular localization of different proteoforms of Aβ using antibodies towards different amino acid sequences or post-translational modifications of Aβ, including clones 4G8, 6F/3D, unmodified- (7H3D6), pyroglutamylated- (N3pE), phosphorylated-(1E4E11) Aβ and Aβ protein precursor (AβPP), in brain biopsies from 3 iNPH subjects, using immunohistochemistry and light microscopy (LM), light microscopy on semi-thin sections (LMst), and electron microscopy (EM).

**Results:**

In LM all Aβ variants were detected. In LMst and EM, the Aβ 4G8, 6F/3D, and the pyroglutamylated Aβ were detected. The AβPP was visualized by all methods. The Aβ labelling was located extracellularly with no specific signal within the intracellular compartment, whereas the AβPP was seen both intra- and extracellularly.

**Conclusions:**

The Aβ markers displayed extracellular localization when visualized by three assessment techniques, reflecting the pathological extracellular accumulation of Aβ in the human brain. No intracellular Aβ pathology was seen. AβPP was visualized in intra- and extracellularly, which corresponds to the localization of the protein in the membranes of cells and organelles.

## INTRODUCTION

Amyloid-β (Aβ) is one of the hallmark lesions of Alzheimer’s disease neuropathological change (ADNC) [1, 2]. Aβ is a product of amyloidogenic cleavage of amyloid-β protein precursor (AβPP), a protein located in the cell membranes and membranes of the organelles. When AβPP is degraded by β- and γ-secretase, pathological Aβ peptides are released into the extracellular space of the brain, forming Aβ aggregates [3, 4]. During the disease process of AD, the Aβ pathology propagates through the predilected neuroanatomical regions [5]. In parallel to the neuroanatomical progress of the pathology, the Aβ protein undergoes biochemical changes; moreover, it produces Aβ variants with an increased ability to aggregate, further promoting synaptic damage and neurotoxicity [3]. The most studied modification is pyroglutamylation of the N-terminus of Aβ, resulting in pyroglutamylated (py) Aβ variant and phosphorylation of Aβ, most often at serine residue 8, resulting in a phosphorylated (p) Aβ variant [3]. The extent of pyAβ is higher in postmortem (PM) brain tissue of subjects with AD than in controls. Furthermore, when detected in the cerebral cortex, it is associated with the extent of hyperphosphorylated (HP) *τ* pathology and the level of cognitive impairment (CI) [6–9]. The pAβ has been detected in animal models of AD and in PM brains from subjects with AD and is associated with a CI [8–12]. The biochemical changes of Aβ are proposed to occur in a hierarchical manner, i.e., the unmodified Aβ detected at the beginning of the disease, following the formation of the pyAβ variants; at the final stage, when the subjects have CI, the pAβ is detected within the aggregates [8, 9]. Even though the Aβ is predominantly detected in the extracellular compartment, there are studies suggesting that some Aβ variants can exert toxic effects when located within neurons [11–14].

Idiopathic normal pressure hydrocephalus (iNPH) is a neurological condition in the elderly, caused by cerebrospinal fluid imbalance, presenting with gait disturbance, CI, and urinary incontinence [15]. The only treatment option available is a ventriculoperitoneal shunt (VPS) insertion that can reverse the symptoms [15]. In some centers in the world, a diagnostic brain biopsy is obtained from the area of the VPS, i.e., frontal lobe, during the shunt surgery.

To date, no hallmark neuropathological lesions of iNPH have been identified. Noteworthy, a substantial number of iNPH subjects display ADNC in their brain tissue [16–18]. When present, Aβ in particular, it is associated with a worse shunt response and progress into AD [17, 19]. According to the international consensus criteria, when seen in the frontal cortex, as in subjects with iNPH, the Aβ pathology reaches low to intermediate level of ADNC [1, 2]. In our previous study, we could illustrate that the biochemical changes seen in the Aβ aggregate composition in AD are also detected in a stepwise manner in iNPH [20]. A single study demonstrated that Aβ can be detected intracellularly in a subject with iNPH [13].

The main assessment of a tissue sample, both for routine diagnostics and research, is performed by light microscopy (LM), applying histochemical- and immunohistochemical (IHC) stainings. The methods are well established and applied all over the world, and the factors altering the outcome are identified [21, 22]. The IHC stainings can be nuclear, membranous, or cytoplasmic; however, even if specific, the ultrastructure cannot be studied in detail. To be able to study the ultrastructure on a cellular level, electron microscopy (EM) is preferred. EM enables immunolabelling (immuno-EM) and makes it possible to study the localization of different proteins within specific cellular compartments [23, 24].

The objective of this study was to assess the cellular compartmentalization of different variants of Aβ using LM and EM on diagnostic surgical brain biopsies obtained during curative VPS insertion from iNPH subjects. This study is unique as it gives a possibility to study human brain tissue, where the confounding factors, such as PM delay and long fixation time, are eliminated. Thus, the results are based on what is seen in a living human with ongoing Aβ pathology.

## MATERIAL AND METHODS

### Ethical approval

The study has been approved by the regional Ethical Committee of Uppsala, Sweden #2013/176, updated 2016; the subjects included have given their informed consent for the use of diagnostic tissue for scientific purposes.

### Study subjects

The study was carried out on surgical brain biopsies from patients diagnosed with iNPH, who underwent a curative VPS surgery at Uppsala University Hospital. During 2018 to 2019, a tissue sample was collected for the EM, in addition to the diagnostic biopsies from 12 subjects.

The biopsies were taken from the area of the shunt channel, i.e., the right frontal lobe, specifically the area of the superior- and the medial right frontal gyri. For LM, the biopsies were fixed in 10% buffered formalin (4% formaldehyde) for 24 h at room temperature. For EM, the biopsies were immediately fixed in 4% paraformaldehyde in 0.1 M phosphate buffer (pH 7.4), then divided into smaller pieces and stored in the paraformaldehyde solution at 4°C until further processing.

### Handling for conventional LM

The tissue samples were processed into paraffin blocks (Histowax from Histolab) and sectioned into 4μm thick sections that were placed on Super Frost slides for Hematoxylin-Eosin stain and Super Frost Plus slides for IHC stainings.

The IHC stainings were performed using an automatic platform, Dako Autostainer Plus with Dako EnVision Flex detection system, according to manufacturer’s instructions. The antibodies (Abs) used and the pretreatments applied are given in Table 1.

**Table 1:** Antibodies for immunohistochemical stains for light microscopy

Antibody	Clone	Company/Code	Dilution	Pre-treatment
Aβ aa17–24	4G8	Biolegend/800703	1:4000	FA 5min
Aβ aa8–17	6F/3D	Dako-Agilent/M0872	1:50	FA 5min
pyAβ N3pE	polyclonal	Tecan/JP18591	1:50	FA 5min
pAβ S8	1E4E11	Kerafast/EBN001	1:500	FA 3min
umAβ S8	7H3D6	Kerafast/EBN002	1:1000	FA 5min
AβPP	22C11	Milipore/MAB348	1:8000	High pH (pH 9.0)
Hyperphosphorylated (Ser202/Thr205) *τ* (TAU8)	PHF-TAU-AT8	Fisher Scientific-Invitrogen/MN1020	1:1000

Aβ, amyloid-β; py, pyroglutamylated; p, phosphorylated; S8, serine residue 8; um, unmodified; AβPP, amyloid-β protein precursor; FA, formic acid 100%.

The samples were assessed at x20 to x400 magnification with Olympus BX45 microscope. All samples were assessed regarding the Aβ 4G8 and HP*τ* pathology. The expression of the other Aβ variants and AβPP was only assessed in the samples that displayed representative tissue on the corresponding EM sample. The extent of Aβ pathology within these samples was graded as follows: 0 = no pathology, 1 = low level of pathology, i.e., single to a couple of aggregates; 2 = moderate level of pathology, i.e., scattered aggregates; 3 = high level of pathology, i.e., numerous aggregates within the sample.

### Handling for transmission electron microscopy (TEM)

#### The embedding procedure

The samples were rinsed in 0.1 M Maleate Buffer (pH 7.4) and post-fixed with reduced osmium tetroxide (OsO_4_) for 1 h. In addition, one sample from each patient was left untreated with OsO_4_. After rinsing, the samples were dehydrated in a graded series of ethanol (50%, 70%, 90%, 99.9%) for 10 min per each step. The samples were placed in a 1:1 mixture of London Resin White (LRW) (Ted Pella, Redding, CA, USA) and 99.9% ethanol for 60 min. Subsequently, the samples were transferred to gelatin capsules with pure LRW left at 4°C overnight. The following day, the capsules were sealed, placed in an oven and the resin was polymerized at 55°C for 48 h.

### Immunogold labelling

#### Light microscopy semi-thin sections

Semi-thin sections (800 nm) were cut using a diamond knife in a Leica UC7 ultramicrotome and transferred to BIOBOND™ (Ted Pella) treated microscope glass slides.

Sections were treated with 50 mM Glycine for 15 min, followed by a Ready-to-use goat blocking solution (Aurion, Wageningen NL) for 20 min. The sections were washed three times with 0.2% acetylated bovine serum albumin (BSA-C™) (Aurion, Wageningen, NL) buffer and incubated with primary Abs for 90 min at room temperature. Thereafter, the sections were washed with BSA-C™ and incubated with a secondary Ab (goat anti-mouse or anti-rabbit) conjugated with a 12 nm gold colloidal particle (Jackson ImmunoResearch, Cambridge, UK) for 60 min at room temperature. After washing with BSA-C™ and Milli-Q (MQ) water, the sections of the gold particles were silver enhanced according to the R-Gent SE-LM kit protocol (Aurion, Wagenigen, NL). Toluidine blue staining was used to counterstain the cells. The Abs used and the pretreatment applied are provided in Table 2.

**Table 2:** The pretreatment strategies for immunohistochemical stains for semi-thin sections in light microscopy and electron microscopy using the same primary antibodies as listed in Table 1

Primary Antibody	Dilution	Host	Secondary Antibody	Company	Dilution
Aβ aa 17–24	1:300	Mouse	Goat anti-mouse (6 nm EM and 12 nm LM)	Jackson ImmunoResearch	1:100
Aβ aa 8–17	Undiluted	Mouse	Goat anti-mouse (12 nm)	Jackson ImmunoResearch	1:100
pyAβ N3pE	Undiluted	Rabbit	Goat anti-rabbit (12 nm)	Jackson ImmunoResearch	1:100
pAβ S8	Undiluted	Mouse	Goat anti-mouse (12 nm)	Jackson ImmunoResearch	1:100
umAβ	1:50 to 1:300	Rat	Goat anti-rat (12 nm)	Aurion	1:100
AβPP	1:1000	Mouse	Goat anti-mouse (12 nm)	Jackson ImmunoResearch	1:100

Aβ, amyloid-β; py, pyroglutamylated; p, phosphorylated; S8, serine residue 8; um, unmodified; AβPP, amyloid-β protein precursor; EM, electron microscopy; LM, light microscopy.

#### Electron microscopy

Ultrathin sections (50–70 nm) were cut with a diamond knife in a Leica UC7 ultramicrotome and placed on nickel mesh grids (Ted Pella) coated with formvar.

Sections were treated with 50 mM Glycine for 15 min, followed by a Ready-to-use goat blocking solution (Aurion, Wagenigen, NL) for 20 min. The sections were washed three times with 0.2% BSA-C™ (Aurion, Wagenigen, NL) buffer and incubated with primary Abs for 90 min at room temperature. Then, the sections were washed with BSA-C™ and incubated with a secondary Ab (goat anti-mouse or anti-rabbit) conjugated with a 6 nm (4G8) or 12 nm gold (all other Abs) colloidal particle for 60 min at room temperature. The Abs used and the pretreatment applied are shown in Table 2.

After washing with BSA-C™ and MQ, the sections were contrasted with 5% uranyl acetate for 10 min and Reynold’s lead citrate for 2 min and examined at 80 kV in a Tecnai™ G2 transmission electron microscope (Thermo Fisher/FEI Company, Eindhoven, NL). Images were acquired using an ORIUS™ SC200 CCD camera (Gatan Inc., Pleasanton, CA, USA) using the Gatan Digital Micrograph software.

## RESULTS

Biopsy samples were collected for both the LM and EM from 12 subjects, four females and eight men, age range 55 to 83 years, mean±standard error of means 74±2. Five of the subjects displayed Aβ in their biopsy, and three of the subjects displayed HP*τ*. Concomitant pathology was seen in two subjects. Out of the 12 subjects, representative grey matter in the EM sample was identified in five subjects, of which three were with Aβ pathology in their biopsy (two females and a man). Thus, three subjects remained for the final IHC and EM analyses. The demographics, diagnostic pathology outcome seen in LM and cases included for the final EM analyses are listed in Table 3.

**Table 3:** Demographics

Patient	Age (years)	Gender	Light microscopy		Electron Microscopy
			Aβ pathology	HP*τ* pathology	with grey matter
1	83	M	1	1
2	78	M	0	0
3	72	M	0	1
4	71	M	0	0
5	79	M	1	0	1
6	79	M	0	0
7	55	M	0	0
8	79	F	1	0	1
9	74	F	0	0
10	75	F	1	1
11	81	F	1	0	1
12	65	M	0	0

M, male; F, female; Aβ, amyloid-β; HP*τ*, hyperphosphorylated *τ*.

### Conventional light microscopy

**Fig. 1:**
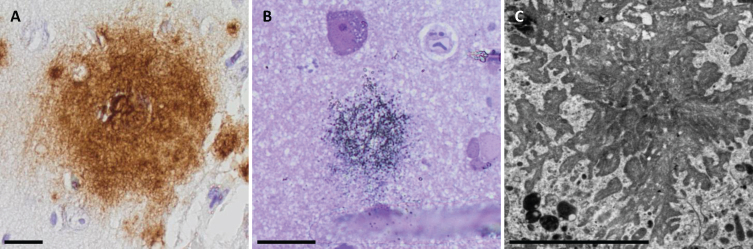
Extracellular amyloid-β in light microscopy (LM), semi-thin sections LM and in electron microscopy. Photomicrographs of extracellular amyloid-β (Aβ) aggregates applying the Aβ 4G8 antibody in LM (A), semi-thin sections in LM (B), and in electron microscopy (C). Bar A. 20μm, B. 20μm, C. 5μm.

High level of extracellular Aβ pathology was visualized in all subjects with Aβ 4G8, 6F/3D and the unmodified (um) 7H3D6 variant (Fig. 1A). The pyAβ (N3pE) was expressed at a high level in two subjects and was moderate in one biopsy. The pAβ (1E4E11) was expressed at a low level in one and at a moderate level in two biopsies. The AβPP was expressed in all samples, both in the neurons and extracellularly.

### Light microscopy semi-thin sections

All subjects displayed immunogold labelled Aβ aggregates when applying the 4G8 antibody in the sample fixed in osmium (Fig. 1B). Two of the subjects displayed the aggregates in their samples without osmium fixation. Due to a lack of grey matter in the sample from the third subject, the sample without osmium fixation was not analyzed. Additionally, Aβ 6F/3D and pyAβ (N3pE) were detected in one subject in a sample without osmium fixation that could not be seen in samples fixed in osmium. The umAβ (7H3D6), pAβ (1E4E11), and AβPP were not detected in semi-thin sections.

### Electron microscopy

In total, there were 12 aggregates detected in all three subjects. The aggregates were star-shaped or in the form of fibrillary streaks in the extracellular compartment. The Aβ fibrils were always located close to the dystrophic neurites (Fig. 1C). However, several dystrophic neurites could be identified within a sample without adjacent Aβ aggregates.

The morphology of the structures was more detailed in the samples fixed in osmium and was more difficult to interpret in the samples fixed without osmium. In contrast, the morphology of immuno-EM samples was more easily visualized in tissue without osmium fixation, as only the Aβ 4G8 Ab was detected to the same extent in samples with and without osmium pretreatment, while the expression of other Abs was sparse or absent in the osmium fixed sample.

The sample without osmium fixation from one subject was lacking the grey matter; thus, the staining was performed only on the osmium fixed sample from this subject.

The Aβ 4G8, 6F/3D and py (N3pE) were detectable using immuno-EM in the two samples without osmium fixation. In the osmium fixed samples, Aβ 4G8 was positive within the aggregates. Additionally, but to a lesser extent, gold particles within the aggregates were detected using pyAβ (N3pE) Ab in two samples and Aβ 6F/3D in one. A few gold particles were seen in the Aβ aggregates in two samples when applying the pAβ (1E4E11) Ab, but single particles were seen in association with the dystrophic neurites and destroyed cells as well. The umAβ (7H3D6) was not detected in any sample.

**Fig. 2:**
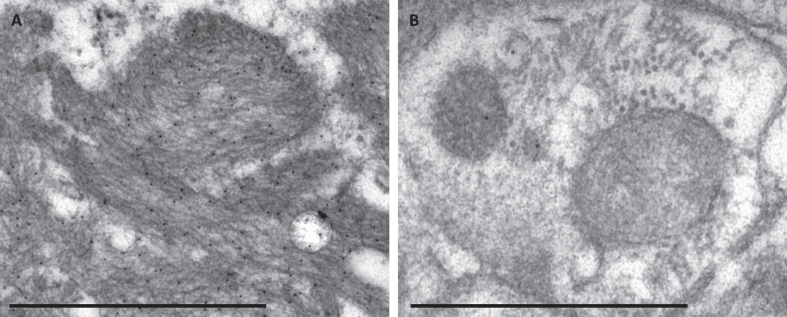
The specificity of the immunogold labelling applying the Aβ 4G8 antibody in electron microscopy. Photomicrograph of specific immunogold labelling, with numerous immunogold particles in extracellular Aβ aggregate (A) and unspecific labelling in a damaged cell structure containing only a few gold particles (B). Bar A. 1μm, B. 500 nm.

The immuno-EM labelling within the extracellular Aβ aggregates was strong and specific (Fig. 2A), containing numerous gold particles for all the Abs except the pAβ (1E4E11), where only a few particles were seen within the aggregate. When applying the Aβ 4G8 and pAβ (1E4E11) Ab, a few gold particles could be seen focally within a dystrophic neurite or a rest of a destroyed cellular structure (Fig. 2B).

The AβPP labelling was sparse and seen in both the intracellular and extracellular compartments.

## DISCUSSION

In this study, we had an opportunity to study the compartmentalization of different biochemical Aβ variants, using LM and EM, in diagnostic brain biopsies from subjects with a clinical diagnosis of iNPH. This is quite unique, as most studies regarding protein compartmentalization in neurodegenerative diseases are performed on PM brain tissue, animal models or cell cultures using IHC or immunofluorescence method [11, 12, 25]. Ultrastructural studies within the topic are few, and there is only one study where the expression of Aβ is seen in a single iNPH biopsy [13, 14].

iNPH is a neurological condition with clinical symptoms of gait disturbance, cognitive impairment and urinary incontinence as well as hydrocephaly on imaging [15]. To date, no neuropathological hallmark lesion of iNPH has been identified in biopsy specimens- or PM studies of subjects diagnosed with iNPH. Nevertheless, a significant number of patients display ADNC in their brain tissue and develop dementia of AD type, over time [16, 26–30].

In line with previous studies, also in this study, ADNC was detected in a substantial number of iNPH subjects, as extracellular Aβ aggregates were seen in five out of 12 biopsies (42%) in LM [16, 26, 27, 29]. The mean age in our study population was 74 years, an age predilected to display neurodegenerative changes in the brain, supporting our results [31, 32]. Moreover, all subjects included for the final analysis displayed an expression of all Aβ markers used, including the umAβ, pyAβ, and pAβ variants. This is interesting, as pyAβ is associated with CI and pAβ with symptomatic dementia in subjects with ADNC and a clinical diagnosis of AD [6–8, 10, 12]. When taking into consideration the stepwise biochemical changes of Aβ aggregate composition, our findings suggest that these iNPH subjects already possess Aβ aggregates typical of symptomatic AD [8].

When assessing the semi-thin sections with the immunogold and toluidine blue staining in the LM, Aβ aggregates were confirmed to be present exclusively in the neuropil, without localization to perikaryal neuronal cytoplasm, in all samples applying the Aβ 4G8 Ab and in one case, applying the Aβ 6F/3D and pyAβ. The umAβ and pAβ were not detected in any sample using this technique.

At the EM level, the Aβ aggregates were seen in the extracellular compartment; furthermore, they varied in shape and were always located next to the dystrophic neurites or damaged cells. This is in line with the neurodegenerative process promoted by ADNC. Aβ pathology is associated with synaptic and neuronal damage and death through different mechanisms, i.e., affecting cell-surface receptors, impairing the neuronal signal transmission, damaging dendritic spines while aggregating Aβ around synapses and cellular processes [33]. When applying the immuno-EM technique, Aβ 4G8, Aβ 6F/3D and pyAβ were detected in all three samples with a strong and specific signal. Numerous gold particles were visualized within the extracellular Aβ fibrils, confirming the extracellular compartmentalization of Aβ with this technique as well. Additionally, a single/few gold particles were seen within a dystrophic neurite or swollen, damaged cell structure when applying Aβ 4G8 Ab, which was interpreted as unspecific background labelling [23].

A few gold particles were seen when applying pAβ within the aggregates and within the dystrophic neurites or destroyed cellular structure in two samples. The very sparse labelling of pAβ in the sample was interpreted as unspecific [23]. The umAβ was not detected in any sample.

AβPP expression was sparse and seen in both the intracellular and extracellular compartments, which corresponds to the localization of this protein in cell membranes and membranes of the organelles [3, 4].

The expression of different proteins varied between the LM samples, semi-thin sections in the LM and immuno-EM, as some proteins were visualized by one technique but not by the others. There are several factors that differ between the techniques used here that can influence the outcome.

The fixative (formaldehyde versus paraformaldehyde), embedding medium (paraffin versus plastic), section thickness (4μm versus 800 nm versus 50–70 nm), antigen retrieval method, choice of Ab and visualization method (chromogen versus gold particle) can all alter the results [21, 23, 24]. Interestingly, a difference was observed in the expression of Aβ 6F/3D and pyAβ between the semi-thin sections with immunogold-silver enhanced staining in LM and what was seen in the EM with the immuno-EM technique. The expression of these Aβ antibodies was only seen in one subject in the semi-thin section sample in the LM, but in all three samples in the immuno-EM. A possible reason is the Abs ability to penetrate the section, as the semi-thin sections are more than ten times thicker than the sections for the EM analysis. Another factor to consider is that the plaque is not present in all sections and could have been sectioned away during preparation.

Two of the antibodies, Aβ 4G8 and pAβ, showed sparse labelling in the form of single particles within the dystrophic neurites and some structures within the swollen, damaged cells. In contrast, Aβ 4G8 expression within the Aβ aggregates was robust, with numerous gold particles observed. The density of labelling seen within the extracellular aggregate, which we interpreted as specific, suggests that the single gold particles seen within the intracellular compartment is unspecific [23]. This can be due to the distance caused by the secondary antibody and the gold particle, as a specific labelling should be seen within 20 nm from the structure of interest [23]. Additionally, Aβ 4G8 is acknowledged to cross-react with AβPP, and the sparse labelling within the swollen cellular structures might represent that cross reaction [34].

We interpreted the very sparse expression of pAβ in the samples as unspecific, as there were only a few gold particles within the plaque structure and only one in an intracellular structure.

Another possible explanation for the discordant observation in the LM and EM is the choice of the Ab used. In immuno-EM, polyclonal Abs are preferred over monoclonal as they recognize multiple epitopes which increase the possibility of surviving the more intensive tissue processing required for EM [23]. This could explain the sparse pAβ expression and total loss of the expression of the umAβ in immuno-EM, since both were visualized by the monoclonal Abs.

To enable this study, an additional biopsy sample for EM was taken from the area of VPS in 12 subjects. Sampling for EM required ethical permit and could be performed on limited number of subjects. In 5 of the 12 biopsies Aβ was seen in LM. Sadly, in only 3 subjects with Aβ pathology in their LM sample, grey matter was present in the EM sample, and thus they could be included for the final EM analysis. The samples are always taken by free hand and thus the sampling is not always optimal. The number of cases in our study is small, which is a weakness, but these samples are unique, and give us an opportunity to study Aβ pathology in a living human.

The biochemical composition of Aβ aggregates and the cellular compartments, where different Aβ variants are exerting their pathological effect, is widely discussed. Intracellular Aβ aggregates have been described in numerous studies, and it is suggested to form toxic oligomers inside the neurons, thus causing neurotoxicity and propagating the pathology through the different neuroanatomical regions [13, 14, 25, 35, 36]. Furthermore, the pAβ associated with symptomatic dementia in AD was also detected intracellularly [12]. Most of the studies assessing this pathology are performed on cell cultures, animal models and PM brain tissue using the IHC or immunofluorescence method. It is worth noting that EM is the method of choice with regard to protein subcellular localization [23]. There are a few studies describing the compartmentalization of Aβ at the EM level, and only one where the pathology is studied in an iNPH biopsy [13, 14]. Unfortunately, the antibodies used in those studies are known to cross-react with AβPP, thus the results should be interpreted with caution [34]. In our setting, we could not visualize specific intracellular Aβ formation or aggregation by any of the markers or the techniques applied.

In conclusion, we had an opportunity to study the compartmentalization of different Aβ markers in surgical iNPH brain biopsies using three different techniques in pathology. In contrast to the majority of previous studies on human brain tissue, we were able to study the pathology in surgical human brain samples where the pre-analytical factors, namely post- mortem delay and post analytical factors, i.e., long fixation time, could be eliminated. All Aβ variants studied were detected in the extracellular compartment, reflecting the pathological extracellular accumulation of Aβ in the human brain. The extracellular labelling was specific and robust with all the techniques used. No specific intracellular Aβ pathology was seen by either of the techniques applied. In diagnostic pathology, the specificity of a staining and the assessment strategy used are of great significance to produce reliable results.

## AUTHOR CONTRIBUTIONS

Sylwia Libard (Conceptualization; Formal analysis; Funding acquisition; Investigation; Resources; Writing – original draft); Monika Hodik (Formal analysis; Methodology; Visualization; Writing – review & editing); Kristina Giuliana Cesarini (Methodology; Resources; Writing – review & editing); Anca Dragomir (Formal analysis; Methodology; Visualization; Writing – review & editing); Irina Alafuzoff (Conceptualization; Formal analysis; Funding acquisition; Investigation; Resources; Writing – original draft).

## Data Availability

The data supporting the findings of this study are available within the article.
